# The Rapid Advance of Dentistry

**Published:** 1894-01

**Authors:** B. Holly Smith


ARTICLE II.

### THE RAPID ADVANCE OF DENTISTRY.

BY DR. B. HOLLY SMITH.

Dentistry has probably made more rapid advancement in the last decade than any other profession. Men skilled in the practice of medicine and surgery have found in this comparatively dewly respectable calling a special field for the application of their knowledge. Trained mechanics and artisans have in it seen opportunity for display of their ingenuity and skill. One invention has seemed to stimulate another inventor until appliances have followed each other so fast and furiously that it would take more time than the busy dentist could afford and more money than he would probably have at his disposal to try all which are brought forward.

The importance of our dental societies cannot be too much emphasized, for it is here we meet and exchange experiences as to appliances and methods, and out of the great mass those which stand the fire of criticism may be selected.

While the improvement along mechanical lines has been marked, there has also been great progress in methods of treatment, the application of new drugs to dental diseases and the introduction of new materials for filling and treatment. There is also a growing feeling among the rank and file of dentists of responsibility as conservators of the organs of mastication, and teeth are no longer ruthlessly extracted, but rather, "How can this tooth be saved?" is asked. With these improvements have come the grateful appreciation and esteem of the public, which has been shown by its willingness to receive education on dental matters from intelligent practitioners. Recognizing the necessity of this education, many dental societies have appointed committees to write articles for the secular press. Much good has been done in the South by the wife of a prominent dentist, who has written a series of letters to mothers upon the care of the teeth of children. This is to me a most interesting branch of the practice of dentistry. If the child is brought to the dentist unprejudiced, it will make a far more desirable patient than many older.

The pernicious habit of nurses and, sad to say, often of mothers, threatening the child with the tortures of tooth extraction creates a feeling of terror and dread of the dentist which is pitiable indeed to see. The importance of preserving the temporary teeth of children cannot be too much emphasized; every child should undergo an examination by a dentist at 2 years and earlier if for any reason decay is suspected; at this time the doctor may judge as to whether the little patient should return every three or every six months. Many parents will say "I do not care to go to the expense of having these teeth filled; they are only temporary teeth; they will soon be lost." To this I would suggest that much of the welfare of the permanent teeth depends upon the preservation in health of these temporary ones and their premature extraction will inevitably entail a long series of irregularities and disease. And besides that, is it nothing that the boy should go supperless to bed for fear of getting something in his tooth to cause pain, or that half the night be spent in suffering, instead of healthful sleep, that his condition of misery should run on until an abscess is formed, the blood impoverished, the health depleted and the child permanently injured? Every dentist sees hundreds of just such cases. Maybe he has a blustering father come into the office, a man who has already, probably, received warning as to his children's teeth, with a "See here, doctor; you must do something with this child's tooth; I was up half the night with him and I am a business man and cannot afford to lose my sleep; pull the tooth out and let's have an end of this." Let me ask this man, has he discharged his obligations to this child which God has given him?

